# Self-Collected versus Clinician-Collected Sampling for Chlamydia and Gonorrhea Screening: A Systemic Review and Meta-Analysis

**DOI:** 10.1371/journal.pone.0132776

**Published:** 2015-07-13

**Authors:** Carole Lunny, Darlene Taylor, Linda Hoang, Tom Wong, Mark Gilbert, Richard Lester, Mel Krajden, Gina Ogilvie

**Affiliations:** 1 Division of STI/HIV Prevention and Control, BC Centre for Disease Control, Vancouver, British Columbia, Canada; 2 University of British Columbia, Vancouver, British Columbia, Canada; 3 Public Health Agency of Canada, Ottawa, Ontario, Canada; 4 BC Public Health Microbiology and Reference Laboratory, Vancouver, British Columbia, Canada; 5 Australasian Cochrane Centre, Monash University, Melbourne, Australia; University of Lausanne, SWITZERLAND

## Abstract

**Background:**

The increases in STI rates since the late 1990s in Canada have occurred despite widespread primary care and targeted public health programs and in the setting of universal health care. More innovative interventions are required that would eliminate barriers to STI testing such as internet-based or mail-in home and community service testing for patients that are hard to reach, who refuse to go for clinician-based testing, or who decline an examination. Jurisdictions such as New Zealand and some American states currently use self-collected sampling, but without the required evidence to determine whether self-collected specimens are as accurate as clinician-collected specimens in terms of chlamydia and gonorrhea diagnostic accuracy. The objective of the review is to compare self-collected vaginal, urine, pharyngeal and rectal samples to our reference standard - clinician-collected cervical, urethral, pharyngeal and rectal sampling techniques to identify a positive specimen using nucleic acid amplification test assays.

**Methods:**

The hierarchical summary receiver operating characteristic and the fixed effect models were used to assess the accuracy of comparable specimens that were collected by patients compared to clinicians. Sensitivity and specificity estimates with 95% confidence intervals (CI) were reported as our main outcome measures.

**Findings:**

We included 21 studies based on over 6100 paired samples. Fourteen included studies examined chlamydia only, 6 compared both gonorrhea and chlamydia separately in the same study, and one examined gonorrhea. The six chlamydia studies comparing self-collection by vaginal swab to a clinician-collected cervical swab had the highest sensitivity (92%, 95% CI 87-95) and specificity (98%, 95% CI 97-99), compared to other specimen-types (urine/urethra or urine/cervix). Six studies compared urine self-samples to urethra clinician-collected samples in males and produced a sensitivity of 88% (95% CI 83-93) and a specificity of 99% (95% CI 0.94-0.99). Taking into account that urine samples may be less sensitive than cervical samples, eight chlamydia studies that compared urine self-collected verses clinician-collected cervical samples had a sensitivity of 87% (95% CI 81-91) and high specificity of 99% (95% CI 0.98-1.00). For gonorrhea testing, self-collected urine samples compared to clinician-collected urethra samples in males produced a sensitivity of 92% (95% CI 83-97) and specificity of 99% (95% CI 0.98-1.00).

**Conclusion:**

The sensitivity and specificity of vaginal self-collected swabs compared to swabs collected by clinicians supports the use of vaginal swab as the recommended specimen of choice in home-based screening for chlamydia and gonorrhea. Urine samples for gonorrhea collected by men had comparably high sensitivity and specificity, so could be recommended as they can be left at room temperature for several days, allowing for the possibility of mail-in home-based testing. In populations that may not go for testing at all, do not have the option of clinical testing, or who refuse a clinical examination, self-collected screening would be a good alternative. We recommend that guidelines on how to self-collect gonorrhea and chlamydia urine, vaginal, rectal and pharyngeal specimens be published.

## Introduction

Rates of sexually transmitted infections (STIs) continue to increase worldwide despite STI control efforts [1–3]. Since the late 1990’s, Canada has experienced an upward trend in reported cases of *Chlamydia trachomatis* (114 per 100,000 population in 1997 to 255/100,000 in 2011) [4,5]. Chlamydia rates for young men in British Columbia have doubled since 1999 [5]. *Neisseria gonorrhea* has also increased in Canada (15/100,000 in 1997 to 34/100,000 in 2011) [5,6]. The increases in STI rates have occurred despite widespread primary care and targeted public health programs and in Canada, in the setting of universal health care. Reducing barriers to testing through low threshold clinics and outreach programs, mass treatment programs and online sexual health education and referral services targeted at hard-to-reach populations are strategies that have been implemented [7–9]. These strategies require substantial public investment but without the desired reduction in STI rates at the population level. More innovative interventions are required that would eliminate barriers to STI testing such as home-based, internet, and mail-in screening.

One such innovative intervention is self-screening for STIs, in the home or in the clinic. Several jurisdictions such as New Zealand [10] and some American states [11,12] currently endorse self-collected sampling for STIs, but without the required evidence to determine whether self-collected vaginal samples are equivalent to clinician-collected samples for the detection of chlamydia and gonorrhea. Current evidence in the Unites States is guided by the Centre for Disease Control, which produces a Morbidity and Mortality Weekly Report (MMWR) to provide evidence for disease control programming. The 2014 MMWR report [13] makes a statement recommending vaginal swab self-sampling for detection of chlamydia and gonorrhea based on two individual studies only [14,15]. The MMWR report states that first catch urine collected from women is acceptable for screening, even though it might detect up to 10% fewer infections when compared with vaginal and cervical swab samples [13]. This statement is based on three individual studies only [16–18]. Clearly, a current meta-analysis of pooled studies is needed to provide evidenced-based guidance. A pooled meta-analysis of studies can provide a more precise estimate of the underlying “true effect” than any individual study [19].

One previous meta-analysis was published in 2005 [20] that included 29 chlamydia and gonorrhea studies comparing self-collected urine samples with clinician-collected cervical and urethral samples. This systematic review is old (nucleic acid amplification tests [NAAT]) have changed significantly and combo assays [combination assays testing both chlamydia and gonorrhea simultaneously] were not available) [21–23] and the statistical methods for meta-analysis of diagnostic studies were developed in 2001 and only adopted recently, therefore the methods are out of date [24,25]. Furthermore, this previous systematic review lacks data related to vaginal, rectal or pharyngeal specimens [20].

Pooled evidence is required to determine whether self-collected specimens are as accurate as clinician-collected specimens for detection of chlamydia and gonorrhea. Screening methods for gonorrhea and chlamydia currently involve the collection of cervical, urine, vaginal, urethral samples and a sample collected by a health care professional during an examination is generally considered as the “gold standard”. Therefore, the objective of this review is to compare the accuracy of screening on self-sampled *versus* clinician-collected specimens. More specifically, we assessed clinical accuracy of NAAT-based chlamydia and gonorrhoea tests when patients collected *vaginal*, *urine*, *pharyngeal and rectal* samples themselves compared to when clinicians collected *cervical*, *urethral*, *pharyngeal and rectal* samples (gold reference standard).

## Methods

We registered our study protocol with PROSPERO. Full methodological details can be found in the study protocol [23]. Our study used clinician-collected specimens as the gold reference standard which assumes 100% accuracy. Databases searched included the Cochrane Database of Systematic Reviews, Web of Science, Database of Abstracts of Reviews of Effects, EMBASE, and PubMed/Medline (January 1990 and June 2013). Search terms included, but not limited to: STIs, chlamydia, gonorrhea, screening, testing, self-collected, home sampling, patient-collected diagnostic accuracy, polymerase chain reaction, and sensitivity and specificity. The bibliographies of retrieved articles were also manually searched as well as key journals such as Sexually Transmitted Infections, Sexually Transmitted Diseases and Journal of Clinical Microbiology.

### Inclusion/Exclusion Criteria

We included randomized, quasi-randomized and non-randomized controlled trials, cross-sectional observational studies examining self-collected verses clinician-collected samples for nucleic acid amplification test (NAAT) screening of gonorrhea and chlamydia. Studies were included if they: (a) used a clinician-collected specimen as their reference standard, (b) included both positive and negative test results and (c) used a NAAT platform of: polymerase chain reaction testing (PCR), strand displacement amplification (SDA), transcription-mediated amplification (TMA), or ligase chain reaction (LCR). We excluded studies if: (a) the time period between self- and clinician-collected samples was longer than three weeks (because of potential change in the disease status of the patient), (b) participants were known to be positive for disease before enrolment in the study, (c) any combined test result (e.g. urine and vaginal combined as self-collected), (d) more than 20 percent of included patients dropped out of the study, (e) authors did not reply to our request for additional data, (f) the study did not include a NAAT, (g) anatomical sites were not comparable (see Table 1 in our published protocol for comparable sites) [23], (h) more than one test was used as a the reference standard, (i) the self-collected and clinician-collected samples were not done on the same person, (j) results were not retested, i.e. a confirmatory test was performed. For budgetary reasons we also excluded non-English-based studies.

**Table 1:** Characteristics of studies comparing the accuracy of self-collected compared to clinician-collected samples (n = 21).

Disease											Sample Collection			Sensitivity	Specificity
	Study, year published	Location	Total participants analyzed	Setting of self-collected specimen	Sex (n)	Age in years (mean or range)	Prevalence	Study Design	QUADAS score(max 12)	Device (self-collection/ clinician collection	Specimen (clinician vs self)	NAAT platform	Specific assay(single or combo)		
**Chlamydia**	Beck-Sague et al. 1998	Atlanta, USA	415	Clinic	F	17	26.6%	Cross-sectional	10	Swab/ Cup	Cervix/ urine	PCR	Amplicor(single)	0.90 [0.81, 0.95]	1.00 [0.99, 1.00]
	Berwald et al. 2009	New York, USA	162	Clinic	F	32	6.8%	Cross-sectional	10	Swab	Cervix/ vaginal	PCR	Amplicor(single)	0.91 [0.59, 1.00]	0.99 [0.95, 1.00]
	Domeika et al. 1999	Vilnius, Lithuania	283	Clinic	F	18–35	9.8%	Cross-sectional	11	Swab	Cervix/ vaginal	PCR	Amplicor(singe)	0.80 [0.56, 0.94]	0.98 [0.96, 1.00]
	Freeman et al. 2011	San Francisco, USA	480	Clinic	M	.	1.3%	Cross-sectional	10	Swab	Pharyngeal/ pharyngeal	TMA	AC2(combo)	0.83 [0.36, 1.00]	1.00 [0.98, 1.00]
	Haugland et al. 2010	Bergen, Norway	603	Clinic	F	15–34	11.8%	Cross-sectional	12	Swab/ Cup	Cervix/ urine	SDA	ProbeTec(single)	0.86 [0.76, 0.93]	0.97 [0.95, 0.98]
	Higgins et al. 1998	Manchester, UK	378	Clinic	M	.	13.8%	Cross-sectional	11	Swab/ Cup	Urethra/ urine	PCR	Amplicor-Cobas(combo)	0.89 [0.77, 0.96]	0.99 [0.97, 1.00]
	Hook et al. 1997	Alabama, USA	309	Clinic	F	.	12.6%	Cross-sectional	11	Swab	Cervix/ vaginal	PCR	LCx(single)	0.91 [0.78, 0.97]	0.98 [0.95, 0.99]
	Keane et al. 2007	Cornwall, UK	333	Clinic	F	16–25	15.9%	Cross-sectional	12	Other	Cervix/ vaginal	PCR	Amplicor-Cobas.(combo)	0.96 [0.87, 1.00]	0.98 [0.96, 0.99]
	Puolakkainen et al. 1998	Helsinki, Finland	1015	Clinic	449 (F); 565 (M)	.	4.2% (F); 7.4% (M)	Cross-sectional	8	Swab/ Cup	Urethra/ urine	PCR	Amplicor-Cobas(combo)	UU: 0.89 [0.77, 0.96]	UU: 0.99 [0.97, 1.00]
	Quinn et al. 1996	Baltimore, USA	525	Clinic	F	24	9.6%	RCT	11	Swab/ Cup	Cervix/ urine	PCR	Amplicor(single)	0.85 [0.70, 0.94]	0.98 [0.95, 0.99]
	Roberts et al. 2011	Dallas, USA	2018	Clinic	F	26.9	4.3%	Cross-sectional	10	Swab/ Cup	Cervix/ urine	TMA	AC2(combo)	0.97 [0.90, 0.99]	1.00 [1.00, 1.00]
	Schachter et al. 2005	Multi-centre, USA	1464	Clinic	F	26.1	13.4%	Cross-sectional	10	Swab/ Cup	Cervix/ urine	PCR	AC2(combo)	0.81 [0.75, 0.86]	0.99 [0.98, 0.99]
	Skidmore et al. 2008	Telford, UK	240	Clinic	F	21	10.4%	Cross-sectional	9	Swab	Cervix/ vaginal	PCR	Cobas taqman 48(single)	1.00 [0.85, 1.00]	1.00 [0.98, 1.00]
	Stary et al. 1997a	Vienna, Austria	312	Clinic	F	.	8.3%	Cross-sectional	11	Swab/ Cup	Cervix/ urine	LCR	LCx(single)	0.92 [0.73, 0.99]	0.99 [0.98, 1.00]
	Stary et al. 1998	Vienna, Austria	240	Clinic	308 (F); 240 (M)	NR	7.5% (F); 17.1% (M)	Cross-sectional	10	Swab/ Cup	Cervix/ urine; Urethra/ urine	LCR	LCx(single)	CU: 0.92 [0.73, 0.99]; UU: 0.83 [0,69, 0.93]	CU: 0.99 [0.97, 1.00]; UU: 0.98 [0.95, 0.99]
	Sugunendran et al. 2001	Liverpool, UK	61	Clinic	M	26	33.7%	Cross-sectional	10	Swab/ Cup	Urethra/ urine	PCR	Amplicor-Cobas(combo)	0.94 [0.71, 1.00]	0.80 [0.65, 0.90]
	Tabrizi et al. 1997	Tiwi, Australia	509	Clinic	F	.	3%	Cross-sectional	10	Tampon	Cervix/ vaginal	PCR	Pace-2(single)	0.93 [0.68, 1.00]	0.97 [0.95, 0.99]
	Toye et al. 1996	Ottawa, Canada	379	Clinic	M	27	4.0%	Cross-sectional	12	Swab/ Cup	Urethra/ urine	PCR	Amplicor(single)	0.87 [0.74, 0.94]	1.00 [0.99, 1.00]
	van der Helm et al. 2009	Amsterdam, Netherlands	2100	Clinic	900 (F); 1200 (M)	31.5	9.4% (F); 10.8% (M)	Cross-sectional	12	Swab	Rectal/ rectal	PCR	Amplicor-Cobas(combo)	F: 0.88 [0.79, 0.94]; M: 0.88 [0.81, 0.92];	F: 0.99 [0.98, 0.99]; M: 0.99 [0.98, 0.99]
	van Doornum et al. 2001	Rotterdam, Netherlands	1001	Clinic	503 (F); 498 (M)	<30	12.1% (F); 9.4% (M)	Cross-sectional	10	Swab/ Cup	Cervix/ urine; Urethra/ urine	PCR	Amplicor-Cobas(combo)	CU: 0.78 [0.67, 0.88]; UU: 0.87 [0.74, 0.94]	CU: 1.00 [0.98, 1.00]; UU: 1.00 [0.99, 1.00]
**Gonorrhea**	Freeman et al. 2011	San Francisco, USA	480	Clinic	M	.	7.2%	Cross-sectional	10	Swab	Pharyngeal/ pharyngeal	TMA	AC2(combo)	0.91 [0.75, 0.98]	0.97 [0.95, 0.98]
	Higgins et al. 1998	Manchester, UK	378	Clinic	M	.	8.7%	Cross-sectional	11	Swab/ Cup	Urethra/ urine	PCR	Amplicor-Cobas(combo)	0.94 [0.80, 0.99]	0.99 [0.98, 1.00]
	Hook et al. 1997	Alabama, USA	309	Clinic	F	.	14.2%	Cross-sectional	11	Swab	Cervix/ vaginal	PCR	LCx(single)	0.98 [0.88, 1.00]	0.97 [0.94, 0.99]
	Schachter et al. 2005	Multi-centre, USA	1464	Clinic	F	26.1	5.5%	Cross-sectional	10	Swab/ Cup	Cervix/ urine	PCR	AC2(combo)	0.88 [0.78, 0.94]	1.00 [0.99, 1.00]
	Stary et al. 1997b	Vienna, Austria	325	Clinic	125 (F); 200 M	.	15.2% (F); 24.5% (M)	Cross-sectional	12	Swab/ Cup	Cervix/ urine; Urethra/ urine	LCR	LCx(single)	CU: 0.89 [0.65, 0.9]; UU (F): 1.00 [0.79, 1.00]	CU: 0.98 [0.93, 1.00]; UU (F): 0.98 [0.94, 1.00]
	van der Helm et al. 2009	Amsterdam, Netherlands	2100	Clinic	900 (F); 1200 (M)	31.5	1.9% (F); 6.9% (M)	Cross-sectional	12	Swab	Rectal/ rectal	PCR	Amplicor-Cobas (combo)	F: 0.85 [0.55, 0.98]; M: 0.88 [0.78, 0.95]	F: 1.00 [0.99, 1.00]; M: 0.98 [0.97, 0.99]
	van Doornum et al. 2001	Rotterdam, Netherlands	1001	Clinic	503 (F); 498 (M)	<30	1.2% (F); 4.2% (M)	Cross-sectional	10	Swab/ Cup	Cervix/ urine; Urethra/ urine	PCR	Amplicor-Cobas(combo)	CU: 0.32 [0.13, 0.57]; UU: 0.84 [0.64, 0.95]	CU: 0.99 [0.98, 1.00]; UU: 1.00 [0.98, 1.00]

Note. F = Female; M = Male; CC = Cervix/ urine; UU = Urethra/ urine.

### NAATs

NAAT testing for the detection of chlamydia and gonorrhea has become the standard diagnostic method used in most laboratories. NAATs have high sensitivity and specificity for the detection of chlamydia and gonorrhea using genital tract samples or first-catch urine specimens [22]. The different NAAT platforms include: PCR (Abbott LCx and Roche Amplicor), SDA (Becton Dickinson ProbeTec ET), TMA (Aptima, GenProbe Aptima Combo 2 [AC2], and Aptima Neisseria gonorrhea assay), and LCR (Abbott LCx [LCx] assay) [22,26,27]. The LCx assay uses LCR amplification and was discontinued in 2002. Combination assays which test for chlamydia and gonorrhea include the AC2, Becton Dickinson ProbeTec chlamydia/ gonorrhea, Amplicor-Cobas, and Abbott Realtime chlamydia/ gonorrhea assays [26,28,29]. The GenProbe PACE 2, discontinued as of December 2012, and the Digene Corporation Hybrid Capture 2 are non-amplified probe tests [30].

### Data Collection

The following data elements were extracted and if appropriate, used for subgroup analysis *a priori*: true positive (TP) false positive (FP); true negative (TN); false negative (FN); sensitivity; specificity; reference (gold) standard used to compare test characteristics; NAAT platform; assay type; specimen type (urine, vaginal, cervical, urethral, pharyngeal, or rectal); gender; mean age; sample size; HIV status; date of the study initiation; country location of study; location of self-collection (home, clinic, ect…); and prevalence. Data was extracted independently by two reviewers using a standardized pre-tested form. Any disagreements between the reviewers were resolved by a third party reviewer. Our main outcomes were the sensitivity and specificity of specific comparable anatomical sites. When comparing anatomical site for comparison, we took into account the fact that cervical samples may be more sensitive than urine or vaginal samples.

### Quality Assessment

Validity was assessed using the Quality Assessment of Diagnostic Accuracy Studies (Quadas) checklist [31]. We adapted the 14 point Quadas checklist to a 12 point checklist. We removed two questions related to the description of the index and reference tests (as chlamydia and gonorrhea tests are well known and described in past literature) and one question related to blinding of the index test patients (as our inclusion criteria stipulated that both tests be conducted on the same patient). We then added one question: Were all included patients counted when reporting true positives, true negatives, false positive and false negatives, and were less than 20 percent of the patients missing from the analysis?

### Data Analysis

TP, FP, TN, and FN from each study were extracted to construct two-by-two tables and test sensitivity and specificity were calculated. Meta-analyses were conducted according to the Cochrane Collaboration’s new recommended methodology for systematic reviews of Diagnostic Test Accuracy [32]. Several studies identified for inclusion in the review had multiple study units (arms) [33]. In these cases we included only one study arm per subgroup to avoid creating unit-of-analysis error. The summary estimates of sensitivity and specificity estimates and their 95% confidence intervals (CIs) were constructed using Stata 11’s metandi Hierarchical Summary Receiver Operating Characteristic (HSROC) model, based on the bivariate normal model for the logit transforms of sensitivity and specificity [25,34,35].

HSROC plots were not fitted if there were four studies or less, as this is not enough to fit the hierarchical models with all five parameters by maximum likelihood. When four studies or less were included, the Moses-Littenburg fixed effects method was used. The area under an ROC was used to pool studies using the fixed effect method providing a summary diagnostic estimate of test accuracy.

Summary estimates were calculated separately for each NAAT platform type (PCR, LCR, TMA, and SDA) or assay type, combination (combo)- or single-disease assay, and other *a priori* categories (prevalence, Quadas score, gender, sample size or device used for sample collection). Sensitivity analyses were conducted to investigate the effect of outliers on the summary estimates. If a disease category contained ten studies or less, meta-regression was not done. Forest plots were generated using RevMan 5.3 [36].

## Results

### Search Results

**Fig 1:**
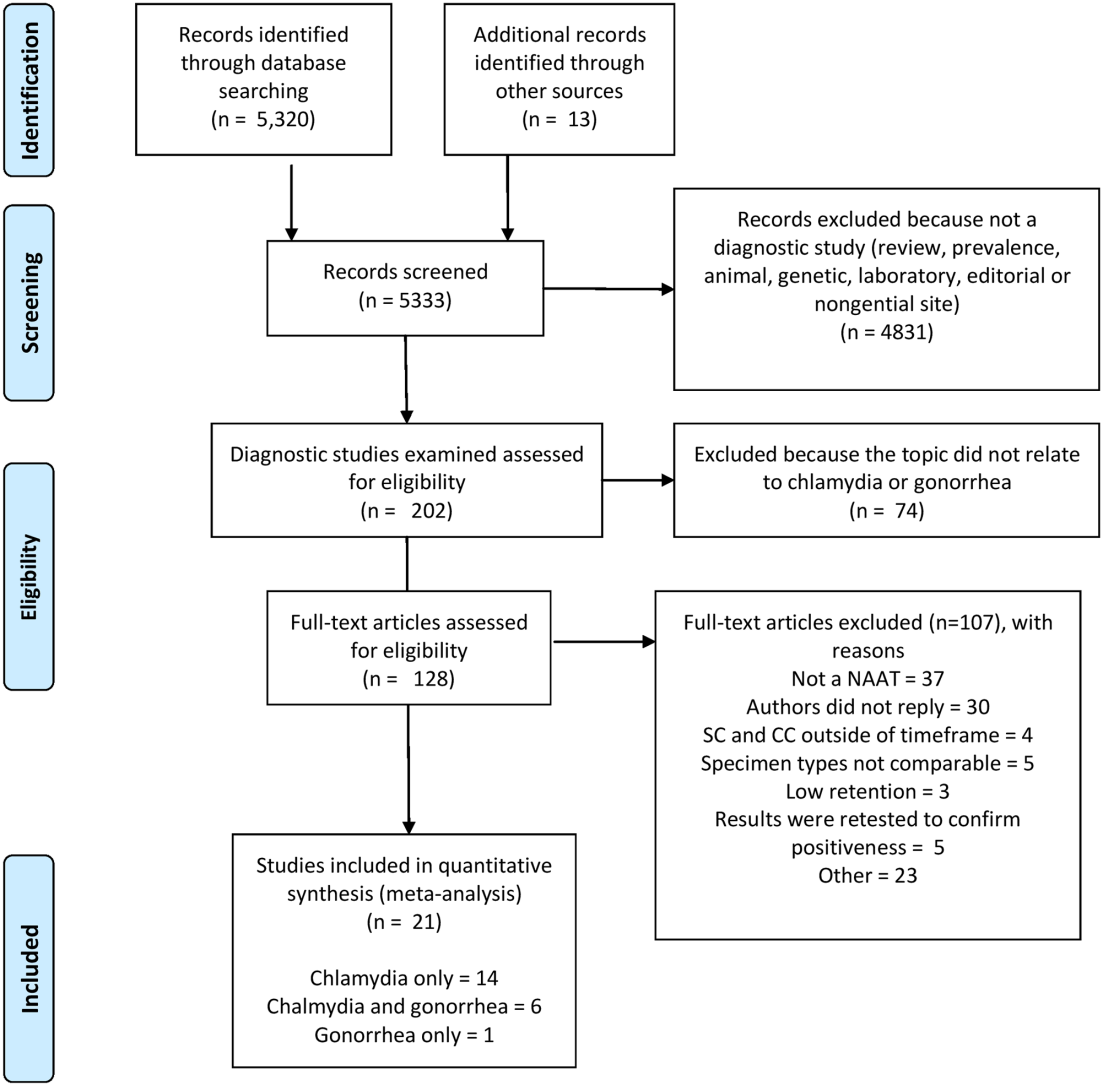
PRISMA flowchart of identification and selection of studies.

A total of 5333 citations were identified (Fig 1). Thirteen references were retrieved from reference lists of identified papers. A total of 4831 titles and abstracts were excluded because they were not diagnostic studies. The remaining 202 citations were retrieved for more detailed evaluation. Of these, 74 were excluded because they were did not relate to the topic. Of the remaining 128 studies comparing self-collected verses clinician collected samples, 107 were excluded (see reasons in Fig 1). Thirty papers were excluded because we could not retrieve the required data from the study and the authors did not reply to our repeat requests for more information. The final number of studies included was 21 (14 conducted chlamydia testing only [37–50], 6 tested for both chlamydia and gonorrhea separately within the same study [17,51–55], and one tested for gonorrhea only [56]. In the14 chlamydia studies, 12,916 participants were included, and in the 6 gonorrhea studies, 6040 participants were included. Table 1 displays the location of the studies, the study designs, patient characteristics, the NAAT platform used and the Quadas score.

### Chlamydia Results

#### Self-collected urine vs clinician-collected urethra samples: Chlamydia in men

Six studies conducted with males compared self-collected urine samples vs clinician-collected urethra swabs (n = 2133) [37,45,47,48,52,55]. These studies were all cross-sectional designs. Sample sizes were relatively similar (range 250–550) except for one very small study of 50 participants [45]. According to the HSROC model, the pooled sensitivity was 0.88 (95% CI 0.83–0.93) and specificity was 0.99 (95% CI 0.94–0.99). All studies had male populations.

In subgroup analysis, one study used LCR as a NAAT platform [37] compared to the other five that used PCR. Two used an assay other than Amplicor-Cobas and used single testing assays [37,47]. Combo assays were used by four studies [45,48,52,55]. When the four combo assay studies were sub-grouped, the sensitivity was 0.90 (0.84–0.94) and specificity was 0.99 (95% CI 0.89–1.00), which was slightly more accurate (Table 2). When the one study with high prevalence (33.7%; [48] was taken out, the sensitivity was 0.89 (95% CI 0.82–0.93) and specificity was 0.99 (0.98–1.00). When Quadas scores were examined, the one study with a score of under 10 was filtered out [48], resulting in a sensitivity of 0.88 (95% CI 0.81–0.92) and a specificity of 0.98 (95% CI 0.93–0.99).

**Table 2:** Pooled effect size of chlamydia studies using a HSROC random effects model (for sub-groups of 4 studies or more).

Self-collected sample/clinician collected sample	Subgroup	Included studies (n)	Sample size (n)	Sensitivity (95% CI)	Specificity (95% CI)
Urine/Urethra in men	All	6	2133	0.88 (0.83–0.93)	0.99 (0.94–0.99)
	Combo assay	4	1833	0.90 (0.84–0.94)	0.99 (0.89–1.00)
	Prevalence (<14%)	5	1583	0.89 (0.82–0.93)	0.99 (0.98–1.00)
	Quadas score (10≥)	5	1583	0.88 (0.81–0.92)	0.98 (0.93–0.99)
Urine/Cervix in women	All	8	6182	0.87 (0.81–0.91)	0.99 (0.98–1.00)
	Single assay	6	4240	0.90 (0.85–0.94)	0.99 (0.98–1.00)
	Prevalence (11%-12%)	4	2853	0.82 (0.78–0.86)	0.99 (0.98–0.99)
	Quadas score (10≥)	4	1638	0.88 (0.81–0.93)	0.98 (0.97–0.99)
	Large to medium sample sizes (500>)	4	4563	0.87 (0.76–0.93)	0.99 (0.97–1.00)
Vaginal/Cervix in women	All	6	1806	0.92 (0.87–0.95)	0.98 (0.97–0.99)
	Single assay	4	1311	0.90 (0.83–0.95)	0.98 (0.97–0.99)
	Device (= swab)	4	400	0.89 (0.82–0.94)	0.98 (0.97–0.99)

**Fig 2:**
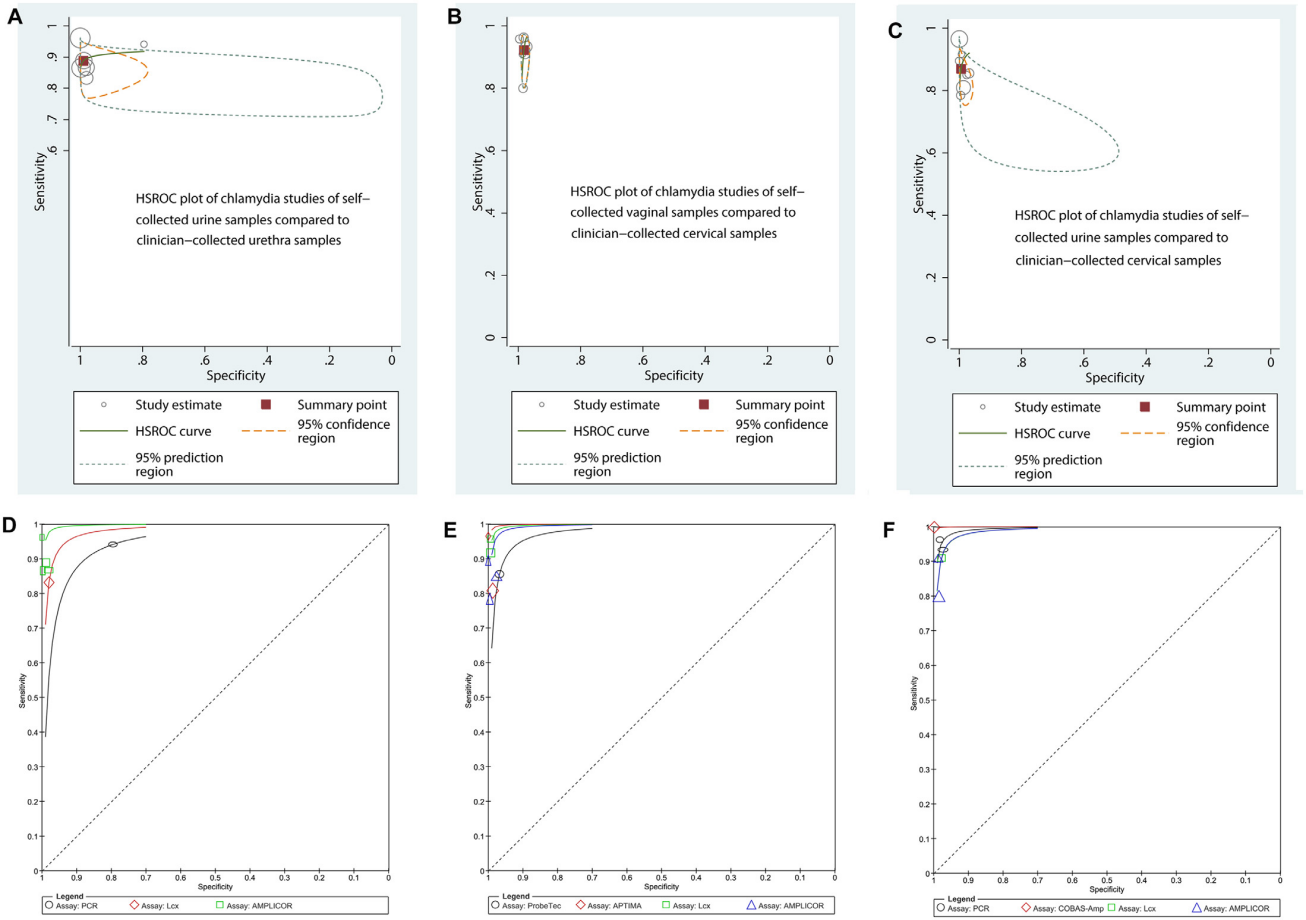
HSROC plots of chlamydia self-collection compared to clinician-collection (top row) and HSROC plots of chlamydia self-sampling by assay type (bottom row). Top row: The circles represent individual studies and size of the circle is proportional to the number of patients included in the study. The red square is the summary estimate of sensitivity and specificity, and the red dotted ellipse around the spots respresents the 95% confidence interval around the summary estimate. The green dotted ellipse around the spots represents the prediction contours outlining the prediction region for the true sensitivity and specificity in a future study. Bottom row: Coloured lines represent the HSROC curve by assay type in Chlamydia studies.

**Fig 3:**
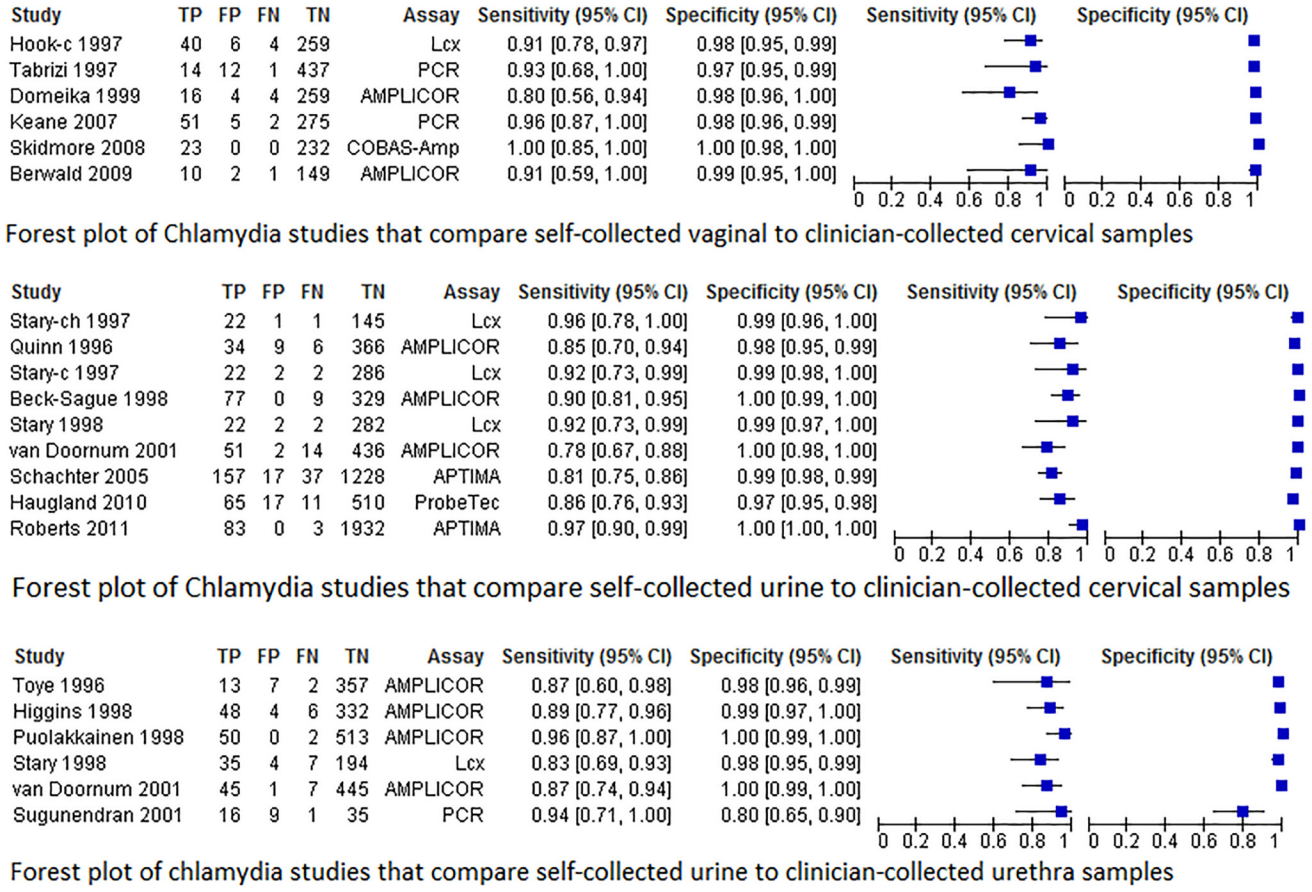
Forest plot of the accuracy of chlamydia self-collection compared to clinician collection.

Fig 2 displays the HSROC curves of the six chlamydia studies of urine self-sampling verses urethra clinician-sampling by type of assay (Amplicor, Amplicor-Cobas or LCx). Forest plots are found in Fig 3. Amplicor-Cobas had a higher sensitivity and specificity than Amplicor or LCx. The tests showed variation in their sensitivity, ranging from 87%-96% in studies using Amplicor-Cobas, 87% for study that used Amplicor, and 83% for LCx. Fig 3 displays the forest plots for chlamydia urine/urethra testing. The funnel plot and the Peter’s test (*p* = 0.17) indicated no publication bias.

#### Self-collected urine vs clinician-collected cervical samples: Chlamydia in women

Eight studies (n = 6182) compared self-collected urine to clinician-collected cervical samples [17,37,38,41,42,49,50,55]. The pooled sensitivity was 0.87 (95% CI 0.81–0.91) and specificity was 0.99 (95% CI 0.98–1.00). These eight were homogenous on sex, age, location where the self-collected swab was obtained and study design (cross sectional except for one randomized control trial; RCT; [42]). Two of these studies used a combo assay [17,55].

In sub-group analysis in studies comparing self-collected urine to clinician-collected cervical samples, sample sizes ranged from large (<1500) to small (>500). When one large study and three medium sized studies were grouped together, the sensitivity was 0.87 (95% CI 0.76–0.93) and specificity was 0.99 (95% CI 0.97–1.00) (Table 2). When the six single assay studies were grouped, the sensitivity was 0.90 (95% CI 0.85–0.94) and specificity was 0.99 (95% CI 0.98–1.00), which was more accurate than all studies combined.

Regarding prevalence, one study had a prevalence of 26.6% [48] and three studies had prevalence of under 10% [37,42,49]. When the four medium prevalence studies were grouped (range 11%-12%), the results for sensitivity (0.82 [95% CI 0.78–0.86]), and specificity 0.99 (95% CI 0.98–0.99) were produced.

When the sole RCT study as well as the three cross-sectional studies with Quadas score of 11 or over [37,41,42,50] were sub-grouped, the sensitivity was 0.88 (95% CI 0.81–0.93) and specificity was 0.98 (95% CI 0.97–0.99).

HSROC plot and the forest plots are found below (Figs 2 and 3). With regard to NAAT assay type, one study used ProbeTec [41], two studies used Amplicor [38,42], one used Amplicor-Cobas [55], two studies used AC2 [17,49], and two studies used LCx [37,50]. Fig 2 shows the HSROC curve of the eight studies of chlamydia by urine self-sampling verses cervical clinician-sampling by type of NAAT (Amplicor, Amplicor-Cobas, AC2, ProbeTec or LCx). AC2 followed by LCx and Amplicor-Cobas had a higher sensitivity and specificity than Amplicor or ProbeTec (Fig 2). The tests showed variation in their sensitivity, ranging from 92% for both studies that used LCx, 81%-97% for AC2, 85%-90% for studies using Amplicor, respectively, and 86% for ProbeTec. No publication bias was detected.

#### Self-collected vaginal vs clinician-collected cervical samples: Chlamydia in women

Six studies comparing self-collected vaginal to clinician-collected cervical samples were included (n = 1806) [39,40,43,44,46,53]. When the studies were pooled, sensitivity was 0.92 (95% CI 0.87–0.95), and specificity was 0.98 (95% CI 0.97–0.99). Studies had sample sizes ranging from 162 to 464 subjects. Two studies did not report mean age of participants [46,53], two had a mean age of 21 years [43,44], and two had recruited participants just over 30 [39,40]. Two out of six studies used a device other than a swab (tampon). All were conducted at the clinic, had Quadas scores of 9 or over, and had prevalence ranging from 3% to 16%. Two studies used a combo assay [39,43]. Of the single-assays, two studies used Amplicor [39,40], one used PCR [53], one used Cobas taqman 48 [44], and one used PACE-2 [46].

In sub-group analysis, grouping four studies that used a swab (excluding those with tampon), resulted in a sensitivity of 0.89 (95% CI 0.82–0.94), and specificity of 0.98 (95% CI 0.97–0.99). When the four single-assay studies were grouped, the sensitivity was 0.90 (95% CI 0.83–0.95) and specificity was 0.98 (95% CI 0.97–0.99), which was less accurate than all studies combined.

HSROC and forest plots are displayed in Figs 2 and 3. All six studies used PCR, so we analyzed the accuracy of the specific PCR assay type (Amplicor, LCx, Amplicor-Cobas or PCR type unknown [noted as simply PCR on the graph]). Fig 2 shows the HSROC curve of the six studies of chlamydia by vaginal self-sampling verses cervical clinician-sampling by type of assay. Cobas taqman 48 had the highest sensitivity and specificity followed by Amplicor-Cobas, and then LCx. Pace-2 or Amplicor (Fig 2). The tests showed variation in their sensitivity, the one study using Cobas taqman 48 had 100% sensitivity, and other studies ranging from 93% to 96% for Amplicor-Cobas, 91% for LCx, and from 80% to 91% for both studies using Amplicor, respectively. No publication bias was detected.

#### Self-collected rectal swabs vs clinician collected rectal swabs: Chlamydia in men and women

Only one high quality cross-sectional study (n = 2312) compared self-collected rectal samples in females and males verses clinician-collected rectal samples [54]. This study was from the Netherlands, had a mean age of 31.5 years, and had a prevalence of approximately 10%. Sensitivity and specificity were stratified by sex. In PCR (Amplicor) testing, female self-collections had a reported sensitivity of 0.88 (95% CI 0.79–0.94) and a specificity of 0.99 (95% CI 0.98–0.99) compared to clinician-collected rectal samples, Male self-collections had a reported sensitivity of 0.88 (95% CI 0.81–0.92) and a specificity 0.99 (95% CI 0.98–0.99).

#### Self-collected pharyngeal swab vs clinician collected pharyngeal swab: Chlamydia in men

Only one US clinic-based chlamydia study (n = 473) compared self-collected pharyngeal samples verses clinician-collected pharyngeal samples [51]. The study population was in low prevalence (1.3%), men who have sex with men (MSM) and the investigators used AC2 as an NAAT assay (TMA). Reported sensitivity was 0.83 (95% CI 0.36–1.00) and specificity was 1.00 (95% CI 0.98–1.00).

### Gonorrhea Results

#### Self-collected urine vs clinician collected urethra samples: Gonorrhea in men

**Fig 4:**
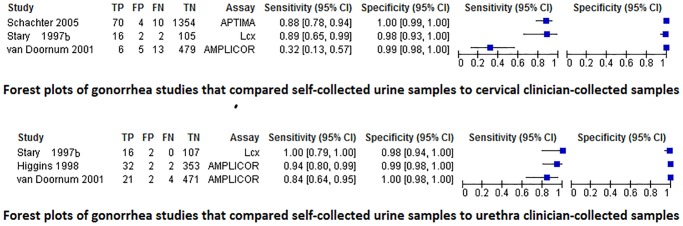
Forest plot of gonorrhea self-sampling compared to clinician sampling.

**Fig 5:**
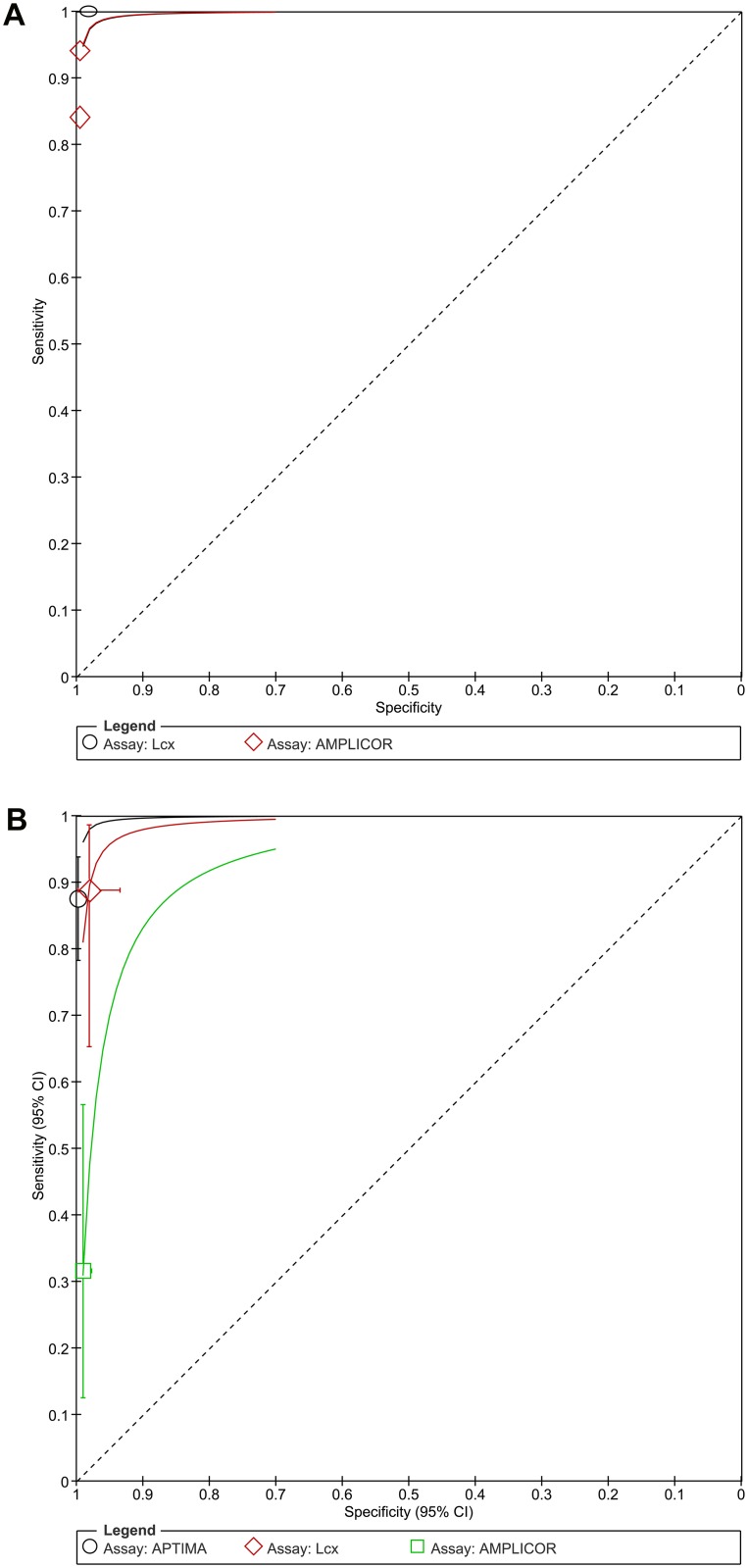
Fixed effects sROC plots of gonorrhea studies comparing self-collected urine to either clinician-collected urethra (left) or cervical (right) samples by PCR assay type. Colored lines represent the sROC curve by assay type.

Three male population studies (n = 1012) comparing self-collected urine samples to clinician collected urethra swabs [52,55,56]. The pooled sensitivity was 0.92 (95% CI 0.83–0.97) and specificity 0.99 (95% CI 0.98–1.00). All were conducted in a clinic setting. Sample sizes were similar and ranged from 125 to 389 participants. They also had similar prevalence (10.6%, 12.1%, and 13.8%, respectively). Quadas scores ranged from 10 to 11. The pooled sensitivity was 0.92 (95% CI 0.83–0.97), and the pooled specificity was 0.99 (95% CI 0.99–1.00), as depicted in the forest plots (Fig 4). Two studies used Amplicor –Cobas [55] [44, 48] and when these were pooled, the sensitivity fell to 0.90 (95% CI 0.79–0.96) and specificity was 0.99 (95% CI 0.99–1.00), and are depicted in the sROC plots (Fig 5). The figure indicates that LCx performed better than the combo assay Amplicor-Cobas studies. No publication bias was present.

#### Self-collected urine vs clinician collected cervical samples: Gonorrhea in women

Three gonorrhea studies compared self-collected urine samples to clinician-collected cervical swabs (n = 2066 women; [17,55,56]). The pooled sensitivity was 0.79 (95% CI 0.70–0.88) and specificity 0.99 (95% CI 0.99–1.00). In all three, the self-collected specimens were conducted at a clinic, had similar prevalence (11%-13%), and similar Quadas scores (10–11). The mean age of all studies was under 30 years. However, the three studies were different in many ways.

They each used a different type of assay, two used combo assays (AC2 and Amplicor-Cobas), and one a single-type assay (LCx). Schachter et al [17] had a large sample size (n = 1438), van Doornum et al [55] had a medium sample size (n = 503), and Stary et al [56] had a small sample size (n = 125). Since the HSROC model requires 4 studies, the fixed effects sROC model was used (Fig 4). Two studies used a combo assay [17,55]. Schachter et al [17] had a large sample size and produced high sensitivity (88%) and perfect specificity (100%). Stary et al [56] also had patients collect urine samples compared to the reference standard, and produced a sensitivity (89%), and specificity (98%). However, the third study under this heading by van Doornum et al [55]) only had 32% accuracy (95% CI 13%-57%) but high specificity (99% [95% CI 98%-100%]). The low accuracy results from Van Doornum et al.’s [55] may be explained by repeated freezing and thawing of specimens as well as their choice of primer with the Amplicor-Cobas assay. No publication bias was present.

#### Self-collected vaginal swabs vs clinician collected cervical samples: Gonorrhea in women

One cross-sectional study (n = 309) compared self-collected vaginal samples to clinician-collected cervical samples was included [53]. The sample collection was by swab and used PCR as a NAAT platform. The reported sensitivity was 0.98 (95% CI 0.88–1.00) and the specificity was 0.97 (95% CI 0.94–0.99).

#### Self-collected rectal swabs vs clinician collected rectal samples: Gonorrhea in men and women

One high-quality cross-sectional study (n = 1626) compared self-collected rectal samples to clinician-collected rectal samples [54]. Both samples were collected using a swab and PCR was used as a NAAT platform. The study stratified their analysis by sex: 697 females and 929 males. For females, the reported sensitivity was 0.85 (95% CI 0.55–0.98) and the specificity was 1.00 (95% CI 0.99–1.00). For males, the reported sensitivity was 0.88 (95% CI 0.78–0.95) and the specificity was 0.98 (95% CI 0.97–0.99).

#### Self-collected pharyngeal swabs vs clinician collected pharyngeal samples: Gonorrhea in men

One cross-sectional study (n = 473) compared self-collected pharyngeal samples to clinician-collected pharyngeal samples [51] among MSM. The sample collection was by swab and a TMA PCR platform was used. The reported sensitivity was 0.91 (95% CI 0.75–0.98), and the specificity was 0.97 (95% CI 0.95–0.98).

### Summary Statement

Fourteen included studies examined chlamydia only, 6 compared both gonorrhea and chlamydia separately in the same study, and one examined gonorrhea. The six chlamydia studies comparing self-collection by vaginal swab to a clinician-collected cervical swab, had the highest sensitivity (92%, 95% CI 87–95) and specificity (98%, 95% CI 97–99), compared to other specimen-types (urine/urethra or urine/cervix). Six studies compared urine self-samples to urethra clinician-collected samples in males and produced a sensitivity of 88% (95% CI 83–93) and a specificity of 99% **(**95% CI 0.94–0.99**)**. Taking into account that urine samples may be less sensitive than cervical samples, eight chlamydia studies that compared urine self-collected verses clinician-collected cervical samples had a sensitivity of 87% (95% CI 81–91) and high specificity of 99% (95% CI 0.98–1.00). For gonorrhea testing, self-collected urine samples compared to clinician-collected urethra samples in males produced a sensitivity of 92% (95% CI 83–97), and specificity of 99% (95% CI 0.98–1.00).

## Discussion

We analyzed the results of 21 studies and over 6100 paired samples to estimate the accuracy of self-collected samples compared to clinician-collected samples for diagnosing chlamydia and gonorrhea. We were able to pool the results of three comparable specimen types by chlamydia and gonorrhea disease. The high sensitivity and specificity of vaginal self-collected swabs compared to swabs collected by clinicians supports the use of vaginal swab self-collection for chlamydia and gonorrhoea testing. However, overall, among the 21 studies included in our analysis, the results of chlamydia and gonorrhea studies indicate acceptable specificity; but sensitivities for both diseases were lower.

For screening studies, we would often prefer to have a high sensitivity and lower specificity. Low sensitivity puts patients at risk of symptomatic infectious disease, and is thus not desired [57]. Low sensitivity may be due to (a) gold standard misclassification, (b) studies conducted in developing countries, (c) collection device used, and (d) cytologic interpretation [57]. In addition, PCR may have a lower sensitivity due to the different primers used in the studies we pooled.

However, if self-sampling is to be used to determine chlamydia status, we suggest self-collected vaginal swabs (compared to the other collection types) as this method has the highest sensitivity (92%) and specificity (98%) among women. In chlamydia tests of female or male urine samples, combo assays performed the best. Urine testing in males is the best option for self-sampling for gonorrhea as the pooled sensitivity was 92% and specificity was 99%.

We included studies that collected samples from the rectum and throat even though these specimen types are not currently approved for NAAT assays, and were used off-label. This has not been reported in previous reviews. However, there were an insufficient number of studies to assess the accuracy of self-collected rectal or pharyngeal samples. We found more studies that evaluated the accuracy of chlamydia self-collected samples compared to clinician collected samples than gonorrhea studies that did the same. As a consequence, we cannot make a recommendation for gonorrhea testing in females as there were not enough studies to pool in female populations.

No clear pattern was apparent which would indicate the accuracy of combo chlamydia/ gonorrhea assays compared to single assays. Nine studies in our sample use a combo assay that tests for both chlamydia and gonorrhea (Amplicor-Cobas or AC2). Twelve studies used an assay that tests for a single STI (LCx, ProbeTec, Amplicor, Cobas taqman 48, or PACE-2). In addition, none of the included studies used the Gen-Probe Aptima test.

Eight chlamydia studies comparing urine self-collected to clinician-collected cervical samples produced a sensitivity of 87% and specificity of 99%, compared to Cook et al.’s [20] results that reported a sensitivity of 83% and specificity of 99%. Our higher findings may be due to the fact that we compared urine self-samples to cervical clinician-collected samples, whereas Cook et al [20] compared urine self-samples to both clinician-collected cervix and urethra samples. In our analysis, AC2 had a higher sensitivity and specificity than LCx (LCR) and Amplicor (PCR). These results compare to Cook’s [20] review which reported the highest summary sensitivities/specificities for urine testing in women in TMA followed by PCR. We therefore recommend TMA tests for urine-based samples.

Six chlamydia studies that used PCR to compare male urine self-samples to urethra clinician-collected samples were pooled and produced a sensitivity of 88% and a specificity of 99%. Again, this is slightly higher than the sensitivities and specificity reported by Cook et al [20] who compared self-collected urine to physician-collected specimens from the cervix and urethra. When we compared the chlamydia screening results of urine-based self-sampling between women and men, we see similar results (87% sensitivity in women compared to 88% in men; and 99% specificity in both gender groups). Cook et al.’s [20] systematic review also found that women and male urine samples had similar accuracy for chlamydia (female sensitivity of 83% compared to male sensitivity of 84%; and specificity 99% for both). Cook’s analysis compared different studies than ours and pooled only three studies compared to our six included studies.

Among the seven gonorrhea studies, three that collected urine compared to urethra samples in males produced a sensitivity of 92% and specificity of 99%, which makes self-collection a viable alternative to physician sampling in men. This is consistent with what Cook et al [20] reported in males (sensitivity of 90% and specificity of 99.7%). In contrast, Cook et al [20] found that the four pooled studies in women had lower sensitivity and specificity of the PCR assay to detect gonorrhea when urine self-samples were taken (56% and 99% respectively).

### Comparison with Other Recommendations

The current MMWR report [13] makes a statement recommending vaginal self-sampling based on two studies only [14,15], both of which we did not include in our results due to poor quality. We excluded the Knox study [14] because a confirmatory test was performed on positive results. We excluded the Masek study [15] as did not have comparative tests and did not perform the self- and clinician-collected samples within one hour of each other. Moreover, the Knox study [14] only had only 318 patients included and the Masek study [15] had swab sample (n = 500) which is small number compared to our meta-analysis. In the Masek study [15], chlamydia detection by self-collection, assayed by ProbeTec and Combo2, had sensitivities of 81% and 100%, with specificities of 100% and 100%, respectively. For gonorrhea detection by self-collection, ProbeTec and Combo2 had sensitivities of 80% and 100%, with specificities of 100% and 100%, respectively. For the Knox study [14], gonorrhea self-collected vaginal swabs had a sensitivity of 72% and specificity of 100%, and gonorrhea self-collected urine sensitivity was 31% and specificity was 100%. Knox et al [14] found that chlamydia self-collected swabs had sensitivity of 85% and specificity of 99%. Chlamydia self-collected urine sensitivity was 73% and specificity was 100%.

For urine self-sampling, the MMWR report [13] states: “First catch urine from women, while acceptable for screening, might detect up to 10% fewer infections when compared with vaginal and endocervical swab samples.” This statement is based on three studies [16–18]. The Falk et al study [18] was excluded from our review because it reported only positive results and the authors could not be contacted with further information. Falk et al [18] reported that the sensitivities calculated in all 171 chlamydia infected women were 88%. Michel et al [16] reported sensitivity of 85%. The Falk and Michel studies did not report specificity. We tried to include the Michel et al [16] study, but the authors did not reply to our request for data. We did include Schachter 2005 data [17] in our meta-analysis [46]. In comparison, our pooled meta-analytic results indicated a good 87% sensitivity in women who collected first catch urine samples.

### Programmatic Considerations

Due to acceptable diagnostic accuracy in our meta-analysis, self-collected specimens could be introduced as a new testing modality in STI control programs for patients that are hard to reach, who refuse to go for clinician-based testing, or who decline an examination. A positive NAAT test for an STI should be followed by a confirmation NAAT. Expanding screening for STIs outside specialist clinical services to internet-based or mail-in home and community service testing can allow more people to get private and anonymous access to testing. If home-based testing involving self-sampling was recommended, sufficient support by STI nurses would have to be provided to patients to properly interpret their test results. Results in a research setting may not translate well in the real world, and thus the introduction of a new screening scenario should be carefully evaluated to the particular context and culture.

One limiting factor that may have impacted the accuracy of patient-collected samples in the included studies is the use (or lack of use) of guidelines on how to self-collect gonorrhea and chlamydia urine, vaginal, rectal and pharyngeal specimens, which may have affected the sensitivities of our pooled results. Some studies such as Freeman et al. [51] and Hook et al [53] gave instructions on self-collection with clear and precise instructions on how to collect a sample, whereas other studies such as Berwald et al. [39] and Keane et al. [43] gave women written instructions, and Puolakkainen et al. [48] did not describe any patient self-collection instructions. Few public health and research organizations have published guidelines about how patients can to properly collect a vaginal or rectal swab specimen [58], a urine sample [59], or a throat swab [60]. The New Zealand guidelines [10] recommend that a self-collected vaginal chlamydia swab may be taken in some circumstances (e.g. annual or opportunistic asymptomatic screening for chlamydia, or when a female declines a genital examination) and that a first catch urine (first 30ml stream) is not the specimen of choice as it has lower sensitivity than vaginal swabs, but is useful if the patient declines examination or to do a self-collected swab. The North Carolina Public Health Program [61] and the Wyoming Public Health Laboratory [12] have written guidelines with instructions for women about how to collect a sample for chlamydia detection. Their instructions state that tampons need to be inserted for at least an hour, and that two consecutive vaginal swabs tipped with Dacron renders better results than using a single one. A John Hopkins guideline [58] for self-collection of a vaginal swab goes through nine steps and stipulates the patient must wash their hands thoroughly, insert the swab one inch inside the vagina, rotate the swab for 15 seconds, remove and not let the swab touch anything, and place the swab in the transport tube. It is clear that public health guidelines are needed that would make the process of self-collection standardized. In addition, studies also did not routinely report on the quality of the samples taken, for example they did not compare the frequency of specimen rejection due to poor sample quality for self-collected specimens vs. clinician-collected rejected specimens.

Self-collected vaginal swabs were the most accurate testing modality in our meta-analysis, and they can be collected in the clinic, at home, in community settings and delivered to a laboratory or sent through the mail. Samples sent through the mail in Canada, through Canada Post, have to be pre-packaged according to their liquid and dry patient specimen guidelines [62]. Swabs can be sent through the mail dry and in a pre-packaged kit. Tampons or sanitary pads can also be used for gonorrhea or chlamydia self-collected testing, but these have not been approved in many jurisdictions [63].

Urine samples had acceptable sensitivity and specificity when collected by patients included in this systematic review for chlamydia/ gonorrhea in men and for chlamydia in women. Urine samples can be left at room temperature for several days, allowing for the possibility of mail-in home-based testing [63]. An alternative to urine collected in a cup has recently been put onto the market. The new sponge-based urine collection device (UriSWAB, Copan Diagnostics, CA, USA) collects urine specimens either by keeping the sponge-tipped applicator under urine stream until saturated, or by dipping the sponge directly into the urine until saturated [64]. In some countries, such as Australia, it is not legal to post liquid biological specimens in the mail; however, UriSwabs are able to be posted, creating opportunities for increased access to testing. STI control programs and clinics aiming to roll-out self-sampling in their populations should routinely evaluate the quality of self-samples taken [65,66].

### Study Limitations

Among the study limitations, several covariates that could affect our summary estimates were not collected, namely: presence of symptoms in patients, and the number of tests done per patient. However, dichotomising patients into asymptomatic/symptomatic was very difficult as no included studies separated the 2x2 table data based on this criteria. When more than one test was done per patient, the first test score was taken as the included data in our analyses, as this was most likely to reduce verification bias. Verification bias occurs when the percentage of subjects receiving disease verification differs according to the test result. Studies that conducted a greater number of diagnostic tests per participant tend to identify more infections (whether correctly or incorrectly), which generally result in a lower calculated sensitivity for the test. We also avoided any possibility of disease re-infection by excluding studies when the time period between self- and clinician-collected sampling was longer than three weeks.

When possible, we grouped studies by prevalence (low, medium or high) to avoid underestimating sensitivity and specificity. However, this was only possible for two chlamydia outcomes (urine self-samples verses urethra clinician samples, and urine self-samples verses cervical clinician samples) and only by one prevalence grouping as there were not enough included studies to compare with other prevalence (low or high) categories. Therefore, we were not able to compared test accuracy by prevalence.

Our study used clinician-collected specimens as the gold reference standard which assumes 100% accuracy. However, 100% accuracy is not a clinical reality. In the physician-collected studies we referenced, sensitivity ranged from about 44% to 99% in gonorrhea NAAT testing depending on the specimen collected, and 33% to 100% in chlamydia NAAT testing depending on the specimen collected [26,67–71]. Another limitation of our meta-analysis was that we included only studies that were published in English, even though included studies were conducted worldwide. Potentially, non-English studies could have changed our reported summary estimates.

In addition, we did not collect unpublished literature, even though papers presented at conferences or done by private test manufacturers may also be applicable. Our justification for this was that unpublished literature would not have been peer-reviewed or generalizable. We also excluded 30 studies because the authors did not reply, which might have biased the results.

There were several gaps in the literature. No studies were included that conducted internet-based (or mail-in) self-collection testing or any studies located in rural areas, as they did not pass our quality assessment for inclusion. There were not enough studies found that conducted gonorrhea self-sampling compared to clinician-based sampling, and this meant that we unfortunately did not have enough studies to pool results from rectal/rectal, vaginal/cervix, or pharyngeal/ pharyngeal specimen gonorrhea groups. In each of the disease and specimen categories, we did not find publication bias which means that we probably found the majority of published studies that examined diagnostic accuracy of self-collected compared to physician-collected chlamydia and gonorrhea samples.

### Elimination of Access Barriers

Uptake of STI testing remains a public health challenge, with particularly low testing rates among young men (age<25) and gay, bisexual and other men who have sex with men (MSM) [72–74]. Other studies and one recent systematic review have shown that self-collection of specimens for STI testing is acceptable among these marginalized populations [11,75–77]. The Odesanmi et al. [76] systematic review found that home-based screening significantly increased the uptake compared to clinic-based testing (RR 1.55, *p*<0.001). Self-sampling can eliminate access barriers to STI testing, such as stigma, shame, negative interactions with service providers, concerns about privacy and confidentiality, inaccessible hours, and travel time to clinics. Furthermore, Gilbert el al. [78]showed that self-collection can be feasibly used within existing community venue-based HIV surveillance systems for MSM.

### Conclusion

The sensitivity and specificity of vaginal self-collected swabs compared to swabs collected by clinicians supports the use of vaginal swab self-collection for chlamydia and gonorrhoea testing. Urine samples for gonorrhea collected by men and women had comparably high sensitivity and specificity, so could be recommended as they can be left at room temperature for several days, allowing for the possibility of mail-in home-based testing. In populations that may not go for testing at all, do not have the option of clinical testing, or who refuse a clinical examination, self-collected screening would be a good alternative. We recommend that guidelines on how to self-collect gonorrhea and chlamydia urine, vaginal, rectal and pharyngeal specimens be published.

The evidence provided in our meta-analysis could help to introduce self-collected specimens as a new testing modality in STI control programs for patients that are hard to reach, who refuse to go for clinician-based testing, or who decline an examination. Expanding screening for STIs outside specialist clinical services to mail-in home-based and community service testing can allow more people to get private and anonymous access to testing.

## Supporting Information

S1 PRISMA Checklist PRISMA 2009 Checklist. (PDF)
